# Predictors of atrial fibrillation recurrence after catheter ablation: a systematic review

**DOI:** 10.1186/s43044-026-00765-0

**Published:** 2026-07-30

**Authors:** Muhanned Faisal Towfig, Mohamed Salah Khalil Salih, Omer Yahia Elhadi Suliman, Mugtaba Ahmed, Selma Ali, Moiad Faisal Suliman Tawfig, Fatima Salah Khalil Salih

**Affiliations:** 1 One Brooklyn Health, Interfaith Medical Center, Brooklyn, NY USA; 2 NYC Health + Hospitals/Harlem Medical Center, New York, USA; 3 Stony Brook University Hospital, Stony Brook, USA; 4 IBN Sina University, Cairo, Egypt; 5 University of Khartoum, Khartoum, Sudan

**Keywords:** Atrial fibrillation, Catheter ablation, Recurrence, Prognostic factors, Risk prediction, Pulmonary vein isolation, Left atrial remodeling, Biomarkers, Machine learning

## Abstract

**Background:**

Recurrence of atrial fibrillation (AF) following catheter ablation is a major clinical problem, and there is considerable variability in patient outcomes. The identification of reliable predictors of AF recurrence is critical in developing patient management strategies.

**Objective:**

To systematically identify and synthesize evidence on independent predictors of atrial fibrillation recurrence following catheter ablation derived from multivariable prognostic models.

**Methods:**

A systematic review was carried out following the PRISMA 2020 guidelines. A comprehensive search was done using PubMed, PubMed Central, Google Scholar, CORE, and DOAJ. Studies were filtered based on the keywords entered with a publication date ranging from January 2016 to January 2026. The tool used for bias assessment was ROBINS-I. Due to the heterogeneity of the data, the results were synthesized narratively, and descriptive statistics were used to measure study characteristics. Conversely, the certainty of the evidence was assessed using the GRADE approach.

**Results:**

Out of the identified 24,083 records, after removing the duplicates, 6817 studies were screened, followed by the collection of 148 full-text articles, and finally, 21 studies were selected for the synthesis after meeting all the inclusion criteria. Advanced age, persistent AF phenotype, increased left atrial size, elevated left atrial pressure, renal dysfunction, frailty, and metabolic abnormalities emerged as the most consistent predictors of AF recurrence. Structural and clinical predictors demonstrated high-certainty evidence. Several biomarkers, including soluble ST2, trimethylamine N-oxide, B-type natriuretic peptide, and insulin resistance indices, were independently associated with AF recurrence, although the certainty of evidence was moderate. Machine learning and radiomics-based models demonstrated promising predictive performance but were limited by insufficient external validation and reduced interpretability.

**Conclusion:**

Atrial fibrillation recurrence after catheter ablation seems to result from the interaction between atrial remodeling, metabolic and inflammation dysfunction, and patients’ clinical features. Conventional predictors based on clinical and structural information seem to be the most robust indicators in terms of prognosis, while novel predictors such as biomarkers and machine-learning approaches require further external validation to be utilized in practice.

**Supplementary Information:**

The online version contains supplementary material available at 10.1186/s43044-026-00765-0.

## Introduction

### Background

Atrial fibrillation (AF) is the most prevalent and clinically relevant form of cardiac arrhythmia. One to two% of people have atrial fibrillation, and this number is likely to rise over the coming years. Recent studies have shown that atrial fibrillation is common among individuals aged 80 and older. AF is more common in men, but rates are equal for men and women as they age. Atrial fibrillation is linked to a higher risk of death, with a 5-fold higher risk of stroke and a 3-fold higher risk of heart failure and hospital stays. It also makes life worse, causes left ventricular dysfunction, and makes it harder to exercise [1–3].

Consequently, enhanced methodologies are necessary to anticipate the complications of atrial fibrillation and facilitate early intervention. At present, the management of atrial fibrillation primarily emphasizes the prevention of thrombosis and the regulation of cardiac rhythm. Catheter ablation (CA) is suggested for symptomatic atrial fibrillation (AF) and diminished left ventricular ejection fraction. In patients with paroxysmal AF, the success rate of CA is 70%, and in those with persistent AF, it is about 50% [4, 5].

The Heart Rhythm Society’s guidelines state that catheter ablation is successful when there are no symptoms or signs of AF, atrial tachycardia, or atrial flutter for at least 30 s after AF ablation. One year of success means that there were no arrhythmic events without antiarrhythmic drugs recorded from the end of the blanking period (which usually lasts three months after ablation) to 12 months of follow-up. Long-term success is defined as the absence of arrhythmic events from the conclusion of the blanking period to a minimum of 36 months of follow-up post-ablation, without the use of antiarrhythmic medications. AF ablation guidelines also included the definition of clinical or partial success as a reduction (≥ 75%) of the AF burden measured with a device that can record ECG tracings or intracardiac electrograms, whether or not antiarrhythmic drugs were present [6, 7].

Early recurrence (ER) occurs when AF develops again within three months of ablation. About half of patients experience this after the procedure. In a consensus among experts on catheter and surgical ablation of atrial fibrillation (AF), early recurrence (ER) is defined as the return of atrial tachyarrhythmia—AF, atrial flutter (AFL), and atrial tachycardia (AT)—within three months following ablation. Many studies have defined this time differently, with some saying it lasts from 1 week to 3 months. This time is often called the blanking period. It has been challenging to come up with a standard definition because of the different blanking periods [8].

Several factors have been recognized as contributors to AF recurrence following CA, including age, duration of AF, ventricular and atrial function, and comorbidities; however, their predictive significance is limited. Thus, catheter ablation (CA) was found to be a key treatment for people with atrial fibrillation (AF) who needed to control their heart rhythm. However, recurrence rates constrain the procedure’s efficacy. Identifying factors associated with ablation success is important for patient selection, optimization of procedural outcomes, and long-term rhythm management [9–12]. Therefore, the aim of this systematic review was to identify, determine, and synthesize current evidence on independent predictors of atrial fibrillation recurrence following catheter ablation among adult patients.

### Objectives

The main objective of this systematic review was to identify and synthesize evidence on independent predictors of atrial fibrillation recurrence following catheter ablation in adults, with a specific focus on predictors derived from multivariable prognostic models. In addition to evaluating the methodological quality and risk of bias of the included studies, to assess the certainty of evidence for each category of predictor, and to compare the predictive performance of traditional clinical and structural predictors with emerging biomarker-based and machine learning–derived prediction models.

Furthermore, this review aimed to give a full narrative synthesis of the most consistently reported predictors and to point out gaps in the current literature in order to support future research and the creation of clinically useful, generalizable prognostic models for everyday use.

## Methods

### Review strategy

This systematic review followed the PRISMA 2020 guidelines and was prospectively planned upon the PICO strategy (Table 1). This review was not prospectively registered in PROSPERO or any other systematic review registry. Relevant studies were searched for in an electronic database through a systematic search. The databases used for the review are PubMed, PubMed Central, Google Scholar, CORE, and Directory of Open Access Journals.

Apart from searching PubMed and PubMed Central, other sources used for search in order to obtain potentially relevant studies include Google Scholar, CORE, and Directory of Open Access Journals (DOAJ). These sources were selected due to the wide range of academic publications in Google Scholar. It is consistent with the practice of conducting systematic reviews since it is a common practice that all results be sorted by relevance, and then only the first 1,000 results need to be screened since relevance drops drastically after this point, and it is practically impossible to screen all obtained results. The search engine provides results only in English between January 2016 and January 2026. Researchers use controlled vocabulary and free text words to search for a population, condition, and setting of interest. The basic Boolean string used was (“atrial fibrillation” OR AF) AND (“catheter ablation” OR “pulmonary vein isolation” OR “PVI” OR “radiofrequency ablation” OR “cryoballoon ablation”) AND (“recurrence” OR “relapse” OR “outcome” OR “predictors” OR “risk factors” OR “prognosis”). The Boolean operators were used to increase sensitivity without losing specificity. The search strategies were adjusted appropriately for each database.

**Table 1:** PICOT variables for the study

*P*	Adult patients aged 18 years or older diagnosed with atrial fibrillation who underwent first-time percutaneous catheter ablation using radiofrequency or cryoballoon techniques.
I	Percutaneous catheter ablation for atrial fibrillation, including pulmonary vein isolation, is performed using either radiofrequency energy or cryoballoon technology. Only index ablation procedures were considered.
C	Patients who experienced atrial fibrillation recurrence after catheter ablation versus those who maintained sinus rhythm during follow-up.
O	Atrial fibrillation recurrence, defined as documented atrial fibrillation, atrial tachycardia, or atrial flutter episodes lasting more than 30 s after a blanking period, as assessed by electrocardiography, Holter monitoring, event monitoring, or implantable cardiac devices.
T	Studies with a minimum follow-up duration of 12 months, with follow-up periods ranging up to 36 months.

### Inclusion and exclusion criteria

Inclusion and exclusion criteria were established to ensure the selection of the most relevant articles.

The inclusion criteria delineated that the target population consisted of adult patients with atrial fibrillation undergoing initial percutaneous catheter ablation, with atrial fibrillation recurrence serving as the outcome measure. The articles published from 2016 to 2026 were incorporated. The inclusion criteria consisted of articles employing multivariable statistical analysis methods, such as logistic regression, Cox proportional hazards, or machine learning models incorporating multivariable feature selection. At least 12 months of follow-up was necessary. Only observational cohort studies, registry-based studies, prognostic model development studies, primarily freely available studies published in English, and studies in full text were deemed eligible.

The exclusion criteria encompassed studies involving surgical or thoracoscopic ablation, hybrid procedures, redo-only ablation cohorts, or populations with highly specific conditions such as congenital heart disease or rheumatic heart disease. Papers that had an exclusive concentration on just one single biomarker, individual imaging modality, early post-procedure predictor, or validation of already established risk scoring systems without coming up with any new predictors were also excluded. The reasoning behind this decision is that the review sought to narrow down its scope by concentrating on independent predictors based on multivariate predictive modeling systems. Additionally, articles that did not have a follow-up period of at least 12 months were excluded. Finally, we excluded systematic reviews, meta-analyses, narrative reviews, editorials, commentaries, guidelines, protocols, conference abstracts, non-English language research, and any research that did not adequately report the data needed for extraction and synthesis.

### Data extraction

Once the initial search was done, all the downloaded literature was imported into Zotero software, and duplication was removed. The title and abstract screening were carried out. The review focused on the population of adult patients presenting with atrial fibrillation and undergoing first-time percutaneous catheter ablation and presenting with the outcome of atrial fibrillation recurrence. The abstracts of studies that investigated and reported the outcome of atrial fibrillation recurrence, which is documented episodes of atrial fibrillation, atrial tachycardia, or flutter greater than 30 s after a blanking period, using electrocardiography, Holter monitoring, event monitoring, and devices were considered and included in secondary screening. Eligible studies underwent full-text assessment according to the predefined inclusion and exclusion criteria. Next, the studies were evaluated based on the inclusion criteria in the secondary screening phase. The reasons for exclusion of studies were as follows: population not appropriate for inclusion, type of intervention not appropriate for inclusion in the studies, studies conducted on very specific populations because of their high specificity, lack of inclusion of a multivariable prognostic model in studies, studies conducted on single biomarkers or isolated imaging parameters such as inflammatory markers, echocardiographic strain parameters, or electrophysiological parameters, lack of inclusion of constructing comprehensive clinical prediction models in studies, studies conducted on validating existing risk score or composite indices without deriving new predictors, studies conducted on early or post-procedural predictors such as very early recurrence, studies conducted for less than 12 months of follow-up, studies not eligible for inclusion based on their design, studies for which there was insufficient or unclear data, studies not published in English, or studies for which abstracts were missing or unavailable in the full text of the article. The titles and abstracts were screened independently by two reviewers. The potentially eligible studies were retrieved in full text and checked against the inclusion and exclusion criteria. Any disagreements were resolved by discussion until consensus was reached. The details of the studies were then extracted using a predesigned data extraction form in MS Excel. The form captured information on general aspects (author, study title, year of publication, objective) and methods (study design, study setting, type of population), in addition to atrial fibrillation subtype, ablation modality, duration of follow-up, outcome definitions, independent predictors identified, statistical methods used, and reported effect measures. The extracted data were analyzed using STATA software version 16.

### Study selection

Initially, a total of 7,283 records were screened (PubMed = 1360; PubMed Central = 3729; Google Scholar = 1000; CORE = 491; and DOAJ = 703), and 466 duplicates were found. After removal of duplications, 6817 records remained. These 6817 records were initially screened (at the title and abstract level). Most of them were excluded as non-relevant to inclusion criteria (6669 studies. Subsequently, 148 full-text articles were assessed for eligibility. Full-text articles were excluded for reasons including inappropriate population, lack of multivariable prognostic analysis, focus on isolated biomarkers or imaging parameters, insufficient follow-up duration, ineligible study design, or inadequate reporting of required outcomes. Ultimately, 21 studies met all inclusion criteria and were included in the final qualitative synthesis.

**Fig. 1:**
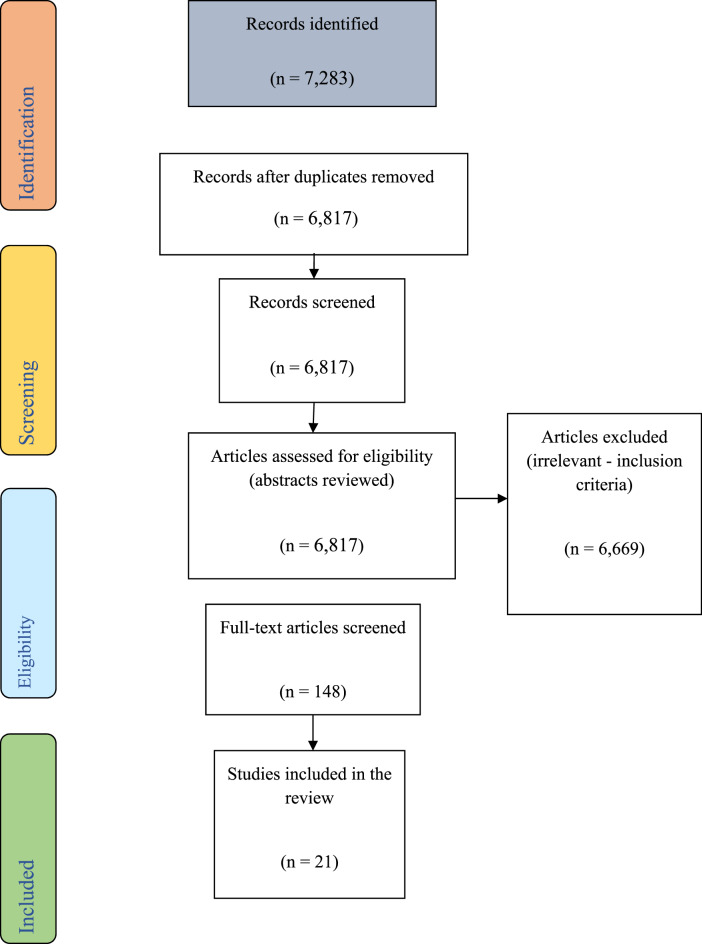
PRISMA Chart of the review

These included 7 studies from PubMed, 6 studies from PubMed Central, 4 studies from Google Scholar, 3 studies from DOAJ, and one study from CORE. Regarding the type of included studies, 13 (61.9%) were retrospective observational cohort studies, 5 (23.8%) were prospective cohort studies or prospective registries, and 3 (14.3%) were prognostic model development studies using advanced statistical or machine learning techniques. The entire study selection process is represented in the PRISMA flow diagram (Fig. 1).

### Quality assessment and risk of bias in the included studies

The final articles included in this review were assessed for quality using ROBINS-I, which is recommended for evaluating methodological quality in observational prognostic research. The assessment covered the following domains: bias due to confounding, bias in the selection of participants, bias in the classification of predictors, bias due to deviations from intended exposures, bias due to missing data, bias in the measurement of outcomes, and bias in the selection of the reported results. Each study was independently evaluated across all ROBINS-I domains and assigned an overall judgment of low risk of bias, moderate risk of bias, serious risk of bias, or critical risk of bias. Studies judged to have a critical risk of bias were excluded from the final synthesis. Discrepancies in bias assessment were resolved through consensus. The risk of bias assessment was used to inform the certainty of evidence evaluation and to guide interpretation of findings in the narrative synthesis [13].

For each study, two reviewers independently performed assessments. Results were summarized in a bias assessment table (Table 2). Due to heterogeneity in the design, interventions, and reported outcomes of studies, meta-analysis was not possible. The results were therefore synthesized narratively, and descriptive statistics were used to measure study characteristics.

**Table 2:** Quality assessment of the included studies

No.	Refs.	Study	Used tool	Quality assessment
1	[14]	Yu et al. 2026	ROBINS-I	Moderate Risk of Bias
2	[15]	Xu et al. 2025	ROBINS-I	Moderate Risk of Bias
3	[16]	Ternes et al. 2025	ROBINS-I	Low Risk of Bias
4	[17]	Lin et al. 2025	ROBINS-I	Moderate Risk of Bias
5	[18]	Jing Ma et al. 2025	ROBINS-I	Moderate Risk of Bias
6	[19]	Jiang et al. 2025	ROBINS-I	Moderate Risk of Bias
7	[20]	Ishiguchi et al. 2025	ROBINS-I	Moderate Risk of Bias
8	[21]	Cui et al. 2025	ROBINS-I	Moderate Risk of Bias
9	[22]	Wang et al. 2024	ROBINS-I	Moderate Risk of Bias
10	[23]	Meng et al. 2024	ROBINS-I	Moderate Risk of Bias
11	[24]	Li RB et al. 2024	ROBINS-I	Moderate Risk of Bias
12	[25]	Gizatulina et al. 2024	ROBINS-I	Moderate Risk of Bias
13	[26]	Wu et al. 2023	ROBINS-I	Moderate Risk of Bias
14	[27]	Sun et al. 2023	ROBINS-I	Serious Risk of Bias
15	[28]	Ilyushenkova et al. 2023	ROBINS-I	Serious Risk of Bias
16	[29]	Li et al. 2022	ROBINS-I	Moderate Risk of Bias
17	[30]	Li et al. 2022	ROBINS-I	Moderate Risk of Bias
18	[31]	Zheng et al. 2021	ROBINS-I	Moderate Risk of Bias
19	[32]	Manfrin M et al. 2021	ROBINS-I	Moderate Risk of Bias
20	[33]	Jia et al. 2021	ROBINS-I	Moderate Risk of Bias
21	[34]	Sultan et al. 2017	ROBINS-I	Low Risk of Bias

### Certainty of evidence assessment

The level of certainty for each identified predictor was assessed using a modified version of the Grading of Recommendations Assessment, Development, and Evaluation (GRADE) approach, specifically for prognostic factor research. The GRADE approach assesses the level of confidence for each identified predictor regarding its association with atrial fibrillation recurrence by evaluating five domains: risk of bias, inconsistency, indirectness, imprecision, and publication bias.

Evidence was initially considered of moderate certainty for observational prognostic studies and was subsequently downgraded or upgraded based on the presence of limitations across these domains. The overall certainty of evidence for each predictor category was classified as high, moderate, low, or very low.

## Results

### Characteristics of included studies

Of the 7283 articles that were retrieved, the final dataset comprised 21 observational studies published between 2017 and 2024. The majority of the studies were retrospective cohort studies, but some were prospective registries or prognostic model development studies. The number of participants in the studies ranged from 150 to over 6000. There were two large national registries: the German Ablation Registry and the Southern Brazilian Registry of Atrial Fibrillation. These are very generalizable in terms of real-world data. All of the studies investigated patients undergoing first-time percutaneous catheter ablation using either radiofrequency or cryo-balloon technology. The majority of the studies investigated patients with both paroxysmal and persistent atrial fibrillation.

The follow-up period of the studies was variable, ranging from 12 months to 36 months. Atrial fibrillation recurrence was defined as documented atrial arrhythmia episodes exceeding 30 s after a blanking period in all of the studies. The monitoring techniques used in the studies were variable and included Holter monitoring, event recorders, and implantable devices (Table 3).

**Table 3:** Characteristics of included studies

Study	Country	Design	Sample size	AF type	Follow-up
Wang et al. [22]	China	Retrospective cohort	1203	Paroxysmal/Persistent	12 months
Wu et al. [26]	China	Retrospective cohort	824	Paroxysmal/Persistent	24 months
Ilyushenkova et al. [28]	Russia	Retrospective cohort	312	Paroxysmal/Persistent	12 months
Li et al. [29, 30]	China	Prospective cohort	1056	Persistent	24 months
Jia et al. [33]	China	Retrospective cohort	687	Paroxysmal/Persistent	12 months
Sultan et al. [34]	Germany	Registry	3679	All types	12 months
Ternes et al. [16]	Brazil	Registry	6178	All types	24 months

### Clinical predictors of atrial fibrillation recurrence

As shown in Table 4, several clinical predictors showed a consistent link to the return of atrial fibrillation across a number of studies. In most large groups, Advanced age was consistently identified as an independent predictor of AF recurrence. Persistent atrial fibrillation phenotype was one of the strongest and most consistent signs that someone would have another episode of atrial fibrillation. Sex was less consistently linked, though some studies found that women had higher rates of recurrence.

There was also a link between recurrence and comorbidity burden. Being frail, having trouble sleeping, having high blood pressure, having kidney problems, or having metabolic syndrome were all linked to worse ablation outcomes on their own. Particularly, researchers often found signs of disease recurrence, such as a lower estimated glomerular filtration rate and signs of insulin resistance. These results suggest that metabolic problems and overall health are important factors in the long-term presence of atrial fibrillation after ablation.

Overall, AF phenotype, patient age, and comorbidity burden were consistently cited in literature as clinical predictors in predicting the outcome. All the predictors had the same direction of effect on outcomes in that they all predisposed to AF recurrences after ablation.

### Structural and echocardiographic predictors

Among the most robust predictors of recurrence were the structural cardiac parameters. Left atrial size, both in terms of diameter and volume, was found to be associated with risk of recurrence in nearly all studies included in the review. Left atrial pressure, as well as left atrial appendage morphology, apart from the windsock configuration, was also found to be associated with risk of recurrence.

In addition, several studies have also reported echocardiographic functional predictors, such as left atrial strain and left atrial appendage function, for recurrence risk post-ablation. These, however, were based on small studies without external validation, making them less useful in comparison to more straightforward structural predictors.

The structural factors exhibited the most consistent results between various studies. The enlargement of left atrial size and the elevation of left atrial pressure were both seen to be predictors of recurrence, with high degrees of certainty among all predictors.

**Table 4:** Main clinical and structural predictors

Predictor	Direction of effect	Consistency across studies	Certainty of evidence
Age	Older age increases recurrence risk	High	High
AF phenotype	Persistent AF increases recurrence	High	High
Left atrial size	Larger LA increases recurrence	High	High
Renal function	Lower eGFR increases recurrence	Moderate	High
Frailty	Higher frailty increases recurrence	Moderate	Moderate
Insomnia	Presence increases recurrence	Moderate	Moderate

### Biomarkers and metabolic predictors

Table 5 summarizes studies evaluating biomarker-based predictors of AF recurrence. Soluble ST2, trimethylamine N-oxide, B-type natriuretic peptide, remnant-like particle cholesterol, and markers of insulin resistance were all independent predictors of AF recurrence after adjustment. These results indicate that AF recurrence is likely influenced by systemic biological processes rather than local cardiac substrate.

Despite the large number of studies on AF, most of the biomarker studies were conducted in small populations. Although all studies showed statistical significance, the confidence intervals were wide, and results were inconsistent.

While there was statistical significance observed between biomarker-based predictors and AF recurrence, the findings were not as convincing as those obtained for clinical and structural factors due to reduced sample size and confidence intervals.

**Table 5:** Biomarkers and advanced prediction models

Predictor/Model	Key study	Association/performance	Certainty
Soluble ST2	Gizatulina et al. [25]	Higher ST2 associated with recurrence	Moderate
TMAO	Meng et al. [23]	Higher TMAO associated with recurrence	Moderate
BNP	Ishiguchi et al. [20]	Higher BNP predicts recurrence	Moderate
Radiomics model	Ilyushenkova et al. [28]	AUC 0.81	Low
XGBoost model	Sun et al. [27]	AUC 0.85	Low

### Machine learning and advanced prediction models

Other studies have utilized machine learning or other advanced statistical modeling methods, such as XGBoost, regularized logistic regression, or radiomics-based models. These models showed superior prediction accuracy compared to conventional models. The area under the curve for these models ranges from 0.70 to 0.85. The machine learning models utilized many predictors. These models demonstrated the ability to identify complex non-linear relationships among predictors and recurrence risk.

Although these machine learning models showed excellent performance, most of them lacked validation. They were conducted on single-center data. Their clinical utility or generalizability is unknown.

### Risk of bias and certainty of evidence

Most of the studies were found to have a moderate risk of bias according to the ROBINS-I tool, mainly because they were retrospective studies with residual confounding and differences in outcome measurements. Compared to smaller studies, large cohorts had a lower risk of bias and better methods. Machine learning and radiomics were similarly linked to a significant risk of bias stemming from challenges related to overfitting and insufficient external validation.

We used a changed version of the GRADE method for prognostic studies to figure out how certain the evidence was. High certainty correlated with structural and clinical predictors, including age, atrial fibrillation phenotype, left atrial size, and renal function. There was a moderate level of certainty linking metabolic and inflammatory biomarkers like ST2, TMAO, and insulin resistance indices. The predictors that used radiomics, advanced imaging, and machine learning were not very certain.

### Overall synthesis of findings

From among all 21 studies, those predictors associated with persistent AF phenotype, increasing age, larger left atrium, high left atrium pressure, kidney dysfunction, and metabolic abnormality were found to be the common predictors of recurrent AF after catheter ablation procedures. Structural and clinical factors were found to exhibit more consistency across the included studies while providing strong levels of evidence. On the other hand, biomarkers and machine learning factors showed promising prediction performance although they had relatively weaker evidence due to their lack of consistency and smaller sample sizes. The results imply that the recurrence of AF was mainly mediated by various factors including structural remodeling, systemic illness, and metabolic problems rather than a particular predictor.

## Discussion

The current systematic review provides an updated and comprehensive overview of current information on predictors of atrial fibrillation recurrence following catheter ablation, including data from 21 studies of high prognostic quality published between 2017 and 2024. Our study confirms that atrial fibrillation recurrence is a multifactorial process resulting from a delicate interaction among various clinical, structural, metabolic, and biological factors. The current studies have identified advanced patient age, presence of a persistent form of atrial fibrillation, increased left atrial size, compromised renal function, and systemic metabolic disorders as the most reliable predictors of atrial fibrillation recurrence. This supports the idea that structural remodeling of the atrium and coexisting systemic diseases are major contributors to long-term failure of catheter ablation.

Our findings are consistent with those reported in the large meta-analysis conducted by Li et al. which looked at over 62,000 patients. They found that the persistence of atrial fibrillation, age, left atrial diameter, and the duration of atrial fibrillation were among the strongest predictors of post-ablation recurrence. For example, the risk of recurrence was 72% higher for patients who had persistent atrial fibrillation, and there were statistically significant dose-response relationships with left atrial diameter and age. The results of this study are very similar to ours. The strongest predictors of a high chance of recurrence after ablation were the persistence of atrial fibrillation and the size of the left atrium. These findings further support the central role of atrial remodeling in AF recurrence. Furthermore, the study by Li et al. found a strong link between early recurrence and long-term failure, which supports our findings about the nature of post-ablation electrophysiological instability as an irreversible substrate for the persistence of atrial fibrillation [35].

The significance of structural remodeling is further reinforced by a study by Zhou et al. who created a clinical nomogram that incorporated early recurrence, maximum left atrial volume index, and parameters of transmitral filling. The study showed excellent predictive ability for late recurrence of atrial fibrillation. The study also reinforced the idea that volumetric burden is a more significant determinant of atrial fibrillation recurrence compared to clinical parameters. Although early recurrence is not considered a predictive factor based on our review, its congruence with structural parameters by Zhou et al. reinforces the primacy of atrial remodeling as a unifying mechanism for catheter ablation failure [36].

Metabolic and inflammatory factors were also identified as key factors that contribute to the recurrence of AF, according to our review results. In fact, various studies included in this review identified insulin resistance indices, renal dysfunction, systemic inflammation, and systemic inflammatory markers such as ST2, TMAO, and BNP as independent predictors of AF recurrence. This is in agreement with the Chinese cohort study by Li et al. which found that obesity, increased levels of C-reactive protein, and increased duration of atrial fibrillation independently predicted AF recurrence within three years of ablation therapy. This suggests that AF recurrence is not an isolated phenomenon confined to the atrium but rather a systemic phenomenon that results from the interplay of various metabolic and inflammatory pathways that promote electrical and structural remodeling of the atrium [37].

The role of heart failure in the development of systemic comorbidities is further emphasized by the systematic review performed by Assayed et al. in which the predictors for atrial fibrillation recurrence in heart failure patients were investigated. Although the population in our review did not include very specific heart failure-only populations, the significant overlap in predictors for atrial fibrillation recurrence in both reviews suggests that the same pathophysiological processes are at play in both the general population and heart failure patients, thus emphasizing the point made in the previous paragraph: atrial fibrillation recurrence is a systemic disease process rather than a procedural outcome [38].

Contrary to conventional clinical predictors, several of the studies in the current review utilized machine learning and advanced statistical modeling for recurrence prediction. These models showed superior discriminative ability compared to conventional models, with area under the curve values close to or greater than 0.80. This finding is consistent with the systematic review and meta-analysis conducted by Fan et al. which showed machine learning models had pooled C-index values of approximately 0.78 in validation datasets. Moreover, convolutional neural network models had superior discriminative ability compared to conventional logistic regression models. Despite excellent performance metrics, most machine learning models in both the current review and Fan et al.’s meta-analysis lacked external validation and utilized highly selected single-center datasets, which raises several concerns about overfitting, interpretability, and generalizability to the general population [39].

Moreover, most machine learning models are “black box” algorithms, which make them difficult to interpret and thus decrease the level of trust clinicians will have in their findings. In addition, there is no consensus about what frameworks for developing models should be used in different research works, which can further limit their comparison and applicability in everyday medical practice. Thus, before routine clinical adoption can be recommended, future machine learning models should undergo rigorous external validation, assessment in diverse patient populations, and evaluation in real-world clinical settings.

Significantly, our results are consistent with the umbrella review by Charitakis et al. which assessed various meta-analyses and found that only a limited number of predictors are associated with a high level of evidence regarding recurrence after ablation. Importantly, time from diagnosis to ablation and persistent AF were found to be among the limited predictors associated with highly suggestive evidence. Other associations, such as hypertension and metabolic disorders, were associated only with suggestive or weak evidence. This is consistent with our certainty of evidence analysis, in which structural and clinical predictors had high certainty, while biomarkers and machine learning-derived predictors were graded down due to methodological limitations [40].

Collectively, our integration with previous meta-analyses and systematic reviews has several important clinical practice implications. First, structural atrial remodeling is again found to be the major predictor of long-term rhythm outcomes following catheter ablation. Second, systemic metabolic and inflammatory factors are found to have a significant and underappreciated role in facilitating arrhythmia persistence. Third, although machine learning has been shown to have favorable predictive performance, it has yet to be externally validated and is also poorly interpreted. Lastly, it is clear that future studies need to focus on externally validated, clinically interpretable, and biologically grounded prognostic models incorporating structural, clinical, and systemic predictors in an integrated risk stratification framework.

In summary, this systematic review is strong evidence for the causes of AF recurrence after catheter ablation since it is determined by a multifactorial model that includes atrial structural remodeling, systemic metabolic disorders, renal impairment, and inflammatory activation. This is a close match with recent meta-analyses and numerous cohort studies, reflecting a paradigm shift towards a more holistic approach rather than a procedure-specific approach to assessing the risks for individual patients. The focus of future research should be on the development of predictive models for catheter ablation in AF.

### Strengths and limitations of the study

This systematic review represents an in-depth and contemporary overview of evidence for predicting AF recurrence after catheter ablation. This review identified consistent evidence supporting the role of structural, clinical, metabolic, and inflammatory factors in AF recurrence following catheter ablation. Among the strongest and clinically significant predictors of AF recurrence after ablation were advanced age, nature of AF, increased left atrium size and pressure, reduced kidney function, frailty, and metabolic disorders, which had moderate to high certainty evidence.

In addition to the traditional clinical and structural predictors, new biomarkers and sophisticated machine learning-based prediction models showed promising predictive accuracy. However, the heterogeneity in methodology, lack of external validation, and decreased interpretability currently impede the clinical use of these methods. Registry-based cohorts and externally validated clinical models provided the most robust and generalizable information.

One limitation in the review is that it did not have prospective registration in the PROSPERO database. While the research was conducted following a preconceived protocol with consideration of the PICO principles and PRISMA 2020, the lack of prospective registration could affect the methodology and overall credibility of the review compared with those that had.

Another limitation relates to heterogeneity in recurrence monitoring strategies across the included studies. Although recurrence was generally defined as atrial arrhythmia episodes lasting more than 30 s after a blanking period, studies differed in surveillance methods, including Holter monitoring, event recorders, and implantable cardiac devices. These differences may have influenced recurrence detection rates and contributed to variability in reported outcomes.

In aggregate, this information points towards a shift towards a combined risk stratification approach that considers structural, clinical, and systemic markers rather than relying on individual markers or procedural characteristics. Future research should be focused on developing and externally validating clinically applicable prognostic models to improve patient selection, ablation strategies, and rhythm outcomes in patients undergoing catheter ablation for AF.

## Conclusion

This systematic review presents a comprehensive and modern perspective of the available literature regarding the predictors used in predicting the recurrence of atrial fibrillation post-catheter ablation. The literature indicates that the predictors of post-ablation AF recurrence are multifactorial in nature, with a significant role being played by structural remodelling in the atria, as well as the effects of clinical burden and underlying metabolic disturbances. Some of the most consistent predictors were older age, persistent atrial fibrillation phenotype, increased left atrial size and pressures, renal impairment, frailty, and metabolic disturbances, with a high to moderate certainty level. These findings should be interpreted within the scope of multivariable prognostic studies included in this review and do not represent the entirety of prognostic evidence related to isolated biomarkers or imaging parameters.

Besides other clinical and structural predictors, novel predictors and machine learning-based predictive models have shown potential for predictive power and biomarkers. These methods, however, have shown limitations because they have shown heterogeneity characteristics that affect interpretation and have failed to show sufficient external validation results beyond a clinical setting. Large registry-based cohorts and clinical models have demonstrated the greatest predictive capability and generalizability results.

Overall, these findings underscore the need for a different approach to risk stratification that considers a patient-centric integrated model, including structural predictors and systemic factors, instead of focusing on isolated predictors and procedural characteristics. Hence, research directions are recommended to address the derivation and external validation of prognostic models to aid in patient optimization, ablation strategy personalization, and eventually post-treatment outcome in AF ablation patients.

## Supplementary Information

Supplementary Material 1. Table S1 summarizes the principal multivariable prognostic models and independent predictors identified across the included studies.

## Data Availability

The datasets generated and analyzed during the current study are available from the corresponding author upon reasonable request.

## Abbreviations

AF: Atrial fibrillation
AFL: Atrial flutter
AT: Atrial tachycardia
CA: Catheter ablation
RFA: Radiofrequency ablation
PVI: Pulmonary vein isolation
LA: Left atrium
LAA: Left atrial appendage
HF: Heart failure
eGFR: Estimated glomerular filtration rate
BNP: B-type natriuretic peptide
ST2: Soluble suppression of tumorigenicity 2
TMAO: Trimethylamine N-oxide
PRISMA: Preferred reporting items for systematic reviews and meta-analyses
PICO: Population, intervention, comparison, outcome
PICOT: Population, intervention, comparison, outcome, time
ROBINS-I: Risk of bias in non-randomized studies of interventions
GRADE: Grading of recommendations assessment, development and evaluation
AUC: Area under the curve
ECG: Electrocardiogram
ML: Machine learning
XGBoost: Extreme gradient boosting
