# Supplementary material for: Predictors of atrial fibrillation recurrence after catheter ablation: a systematic review
Source: Egypt Heart J. 2026 Jul 30;78:60. doi: 10.1186/s43044-026-00765-0 (PMC13424023; doi:10.1186/s43044-026-00765-0)
Supplement: Supplementary file 1 — Supplementary Material 1. Table S1 summarizes the principal multivariable prognostic models and independent predictors identified across the included studies. [file 43044_2026_765_MOESM1_ESM.docx]

# Supplementary Table S1. Principal Multivariable Prognostic Models and Independent Predictors of AF Recurrence After Catheter Ablation.

| **Study** | **Model Type** | **Key Independent Predictors** | **Performance Measure** |
| --- | --- | --- | --- |
| [Yu et al., 2026] | Nomogram | Age, AF type, LA size, clinical variables | Risk nomogram |
| [Xu et al., 2025] | Cox regression | Frailty score | HR reported |
| [Ternes et al., 2025] | Registry analysis | Age, persistent AF, comorbidities | Multivariable model |
| [Lin et al., 2025] | Multivariable regression | Left atrial appendage morphology | Adjusted OR/HR |
| [Jing Ma et al., 2025] | Multivariable regression | H-type hypertension, BMI | Adjusted HR |
| [Jiang et al., 2025] | Nomogram | CHA2DS2-VASc components and clinical variables | Nomogram |
| [Ishiguchi H et al., 2025] | Multivariable regression | BNP levels | Adjusted HR |
| [Cui et al., 2025] | Regularized logistic regression | Sex, AF duration, LA size, clinical variables | Predictive model |
| [Wang et al., 2024] | Multivariable regression | Insulin resistance indices | Adjusted HR |
| [Meng et al., 2024] | Multivariable regression | TMAO level | HR; ROC analysis |
| [Li RB et al., 2024] | Multivariable regression | Insomnia | Adjusted HR |
| [Gizatulina et al., 2024] | Multivariable regression | Soluble ST2 | Adjusted HR |
| [Wu et al., 2023] | Multivariable regression | Remnant-like particle cholesterol | Adjusted HR |
| [Sun et al., 2023] | XGBoost model | Multiple clinical predictors | AUC ≈ 0.85 |
| [Ilyushenkova JN et al., 2023] | Radiomics model | Periatrial adipose tissue radiomic features | AUC ≈ 0.81 |
| [Li RB et al., 2022] | Multivariable regression | Subclinical thyroid dysfunction | Adjusted HR |
| [Li G et al., 2022] | Risk prediction model | Age, AF characteristics, LA parameters | Predictive model |
| [Zheng et al., 2021] | Multivariable regression | Reduced eGFR | Adjusted HR |
| [Manfrin M et al., 2021] | Multivariable regression | Elevated left atrial pressure | Adjusted OR/HR |
| [Jia S et al., 2021] | Logistic regression model | Clinical and echocardiographic variables | Logistic model |
| [Sultan et al., 2017] | German Ablation Registry | Age, AF type, comorbidities | Registry model |
